# Clip-with-line traction-assisted modified double-layered suturing closure for gastric endoscopic submucosal dissection defects

**DOI:** 10.1055/a-2936-2688

**Published:** 2026-08-20

**Authors:** Shun-ichiro Ozawa, Satoshi Wakao, Kenjiro Takeda, Daimon Shirose, Hasegawa Hiroyuki, Mitsuharu Fukasawa

**Affiliations:** 1 Department of Gastroenterology 74164 Japan Community Health Care Organization (JCHO) Yamanashi HospitalKofuYamanashiJapan

Complete closure of gastric mucosal defects after endoscopic submucosal dissection (ESD) is desirable to reduce delayed adverse events. 1​ Although dedicated endoscopic suturing devices have recently been developed, 2​ they require advanced expertise and additional cost. The modified double-layered suturing (ORIGAMI) method 3​ ​4​ enables the inversion of the muscle layer and double-layered closure without dead space; however, closure may be difficult when the defect is viewed tangentially, particularly in the thick gastric wall, because adequate tissue tension cannot be obtained. An 80-year-old man with a 25-mm type 0-IIc intramucosal adenocarcinoma located on the greater curvature of the upper gastric body underwent en bloc ESD. The post-ESD mucosal defect was tangential to the endoscopic view, making complete closure with the modified ORIGAMI method alone technically challenging.

A clip-with-line was prepared according to the method described by Oyama et al 5​ using a reopenable clip (SureClip 16 mm; Micro-Tech Co. Ltd, Nanjing, China) and dental floss and was first placed on the distal muscle layer. External traction applied through the line changed the orientation of the gastric wall ( Fig. 1 ). Sequential approximation of the defect edges was then performed under clip-with-line traction without dead space. Because of the large mucosal defect, an additional clip-with-line was prepared in the same manner and placed on the oral side muscle laye **r** to maintain adequate traction and facilitate complete closure. The procedure is shown in Fig. 2 and Video 1 . Complete closure was achieved using a combination of 16-mm Lockado clips (Micro-Tech Co. Ltd, Nanjing, China) and SureClips, requiring a total of eight clips in 17 minutes, and the traction lines were cut with the same DualKnife J 1.5 mm (KD-655Q; Olympus Medical Systems Corp., Tokyo, Japan) used for the ESD. This technique requires neither dedicated devices nor complex preparation. Endoscopists experienced in clip-with-line traction during ESD 5​ can readily adopt this technique using standard equipment. Clip-with-line traction-assisted modified ORIGAMI closure may represent a simple, reproducible, and cost-effective option for complete closure of gastric post-ESD mucosal defects.

**Fig. 1:**
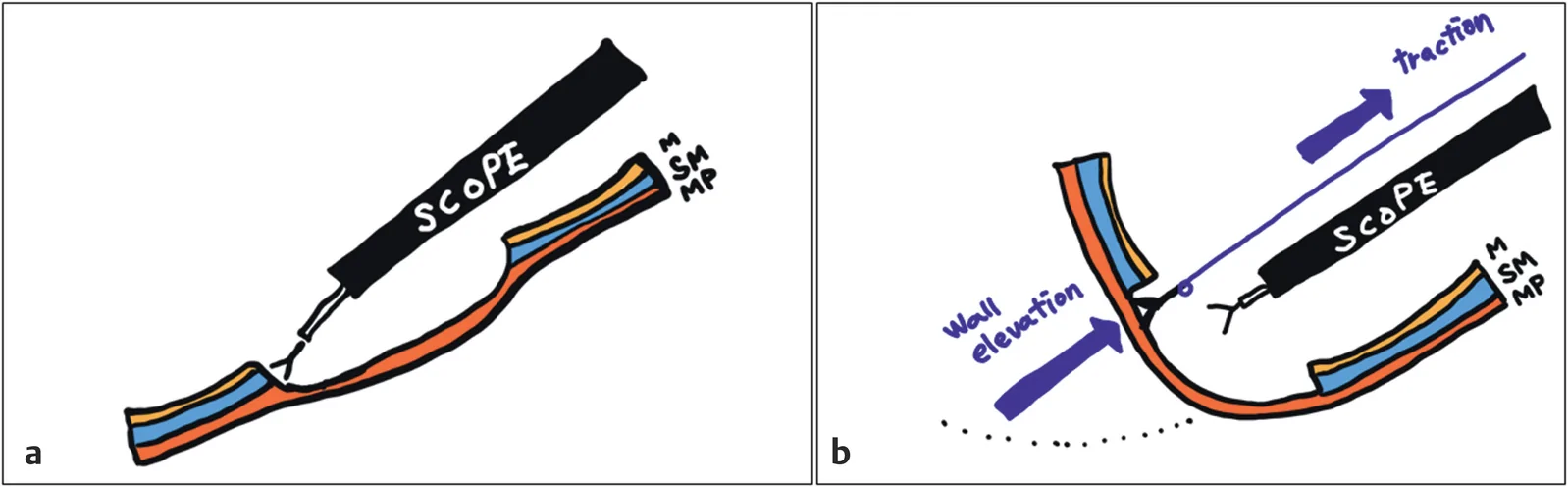
Schematic illustration showing how clip-with-line traction changes the orientation of the gastric wall during modified ORIGAMI closure. ( **a** ) Tangential orientation of the gastric wall before traction. ( **b** ) External traction applied through the line changes the orientation of the gastric wall, facilitating subsequent approximation of the defect edges. M, mucosa; SM, submucosa; MP, muscularis propria.

**Fig. 2:**
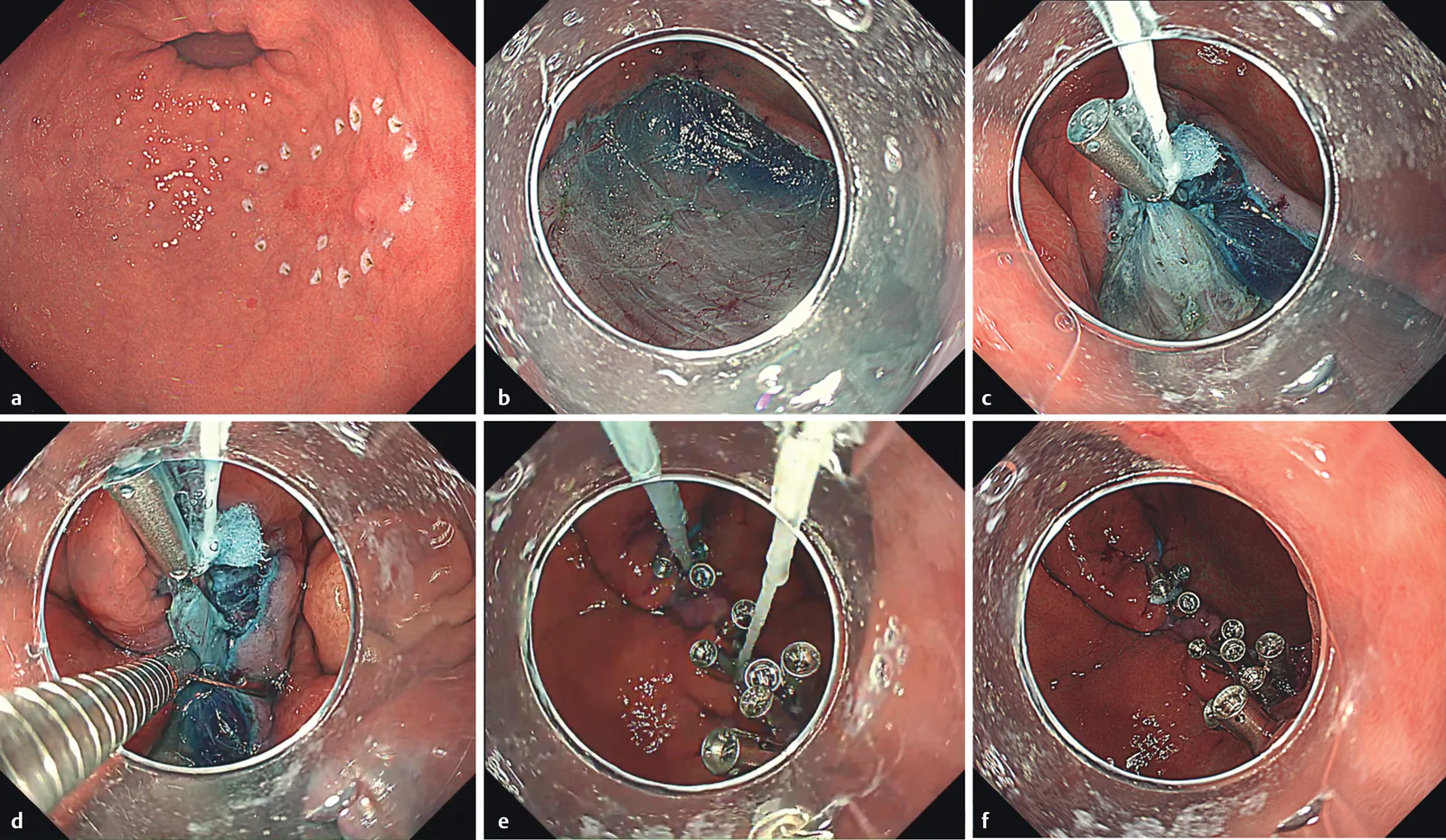
Clip-with-line traction-assisted modified ORIGAMI closure for a gastric post-ESD mucosal defect. ( **a** ) Post-ESD mucosal defect. ( **b** ) A clip-with-line was placed on the distal muscle layer. ( **c** ) External traction applied through the line changed the orientation of the gastric wall. ( **d** ) Sequential approximation of the defect edges under clip-with-line traction. ( **e** ) An additional clip-with-line was placed on the oral side muscle layer to maintain adequate traction and facilitate complete closure. ( **f** ) Complete closure of the post-ESD mucosal defect.

**Video 1** Clip-with-line traction-assisted modified ORIGAMI closure for a gastric post-ESD mucosal defect. A clip-with-line prepared using a reopenable clip (SureClip 16 mm) and dental floss changed the orientation of the gastric wall, enabling sequential approximation of the defect edges and complete closure without dead space using standard equipment. Video-URI: .


Endoscopy_UCTN_Code_TTT_1AO_2AO

## Declaration of GenAI Use

During the writing process of this paper, the authors used ChatGPT (OpenAI) to assist with English language editing and improvement of manuscript readability. The authors reviewed and edited all the AI-assisted contents and take full responsibility for the final content of the manuscript.

## Data Availability

The data are not publicly available because they contain information that could compromise patient privacy but are available from the corresponding author upon reasonable request.
